# Tick-borne Encephalitis Associated with Consumption of Raw Goat Milk, Slovenia, 2012

**DOI:** 10.3201/eid1905.121442

**Published:** 2013-05

**Authors:** Neda Hudopisk, Miša Korva, Evgen Janet, Marjana Simetinger, Marta Grgič-Vitek, Jakob Gubenšek, Vladimir Natek, Alenka Kraigher, Franc Strle, Tatjana Avšič-Županc

**Affiliations:** Regional Public Health Institute, Ravne na Koroškem, Slovenia (N. Hudopisk, E. Janet, M. Simetinger);; Institute of Microbiology and Immunology, Ljubljana, Slovenia (M. Korva, T. Avšič-Županc);; National Institute of Public Health, Ljubljana, Slovenia (M. Grgič-Vitek, A. Kraigher);; University Medical Centre Ljubljana, Ljubljana (J. Gubenšek, F. Strle);; Regional Hospital Slovenj Gradec, Slovenj Gradec, Slovenia (V. Natek)

**Keywords:** TBEV, tick-borne encephalitis virus, goat milk, raw goat milk, Slovenia, viruses

## Abstract

Tick-borne encephalitis (TBE) developed in 3 persons in Slovenia who drank raw milk; a fourth person, who had been vaccinated against TBE, remained healthy. TBE virus RNA was detected in serum and milk of the source goat. Persons in TBE-endemic areas should be encouraged to drink only boiled/pasteurized milk and to be vaccinated.

In Europe, tick-borne encephalitis (TBE) is one of the most common flavivirus infections of the central nervous system and is endemic to several countries. Slovenia is among European countries with the highest reported TBE incidence rates (8.1–18.6 cases/100,000 population in the past decade) (*1*). TBE virus (TBEV) is mainly transmitted by tick bites but occasionally is transmitted by ingestion of unpasteurized milk/milk products from infected livestock (*2*).

Previously, large TBE outbreaks linked to a common source had been associated with consumption of dairy products (mostly goat milk); in recent years, smaller, dairy product–associated outbreaks have been reported from several TBEV-endemic countries (*3*–*6*). Despite high TBE incidence rates and low uptake of TBE vaccine among the Slovenian population (*7*), alimentary transmission of TBEV had not been reported in the country. We report a small outbreak of TBE that occurred in 2012 among persons in Slovenia who consumed raw goat milk.

## The Study

On May 8, 2012, acute symptomatic TBEV infection was diagnosed in a kidney transplant patient in Slovenia (Table, Patient 1). A possible link between the infection and consumption of raw goat milk was revealed, triggering a detailed investigation of possible sources of infection and of 3 other persons who, together with patient 1, had consumed ≈2 L of raw milk (colostrum) from the same goat on April 18 (Table). Two days after the milk was consumed, fever, fatigue, and malaise developed in 3 of the 4 person, including Patient 1, who also had headache and myalgia.

**Table:** Epidemiologic characteristics of persons in whom tick-borne encephalitis developed after drinking raw goat milk. Slovenia, 2012*

Patient no., age, y/sex	Date(s) milk consumed	Illness phase			Date(s) serum sample obtained					
First		Second	Virologic testing				
Onset date; clinical signs; duration		Onset date; clinical signs; duration	TBEV ELISA			TBEV NT	TBEV rRT-PCR
			IgM		IgG		
1, 31/M	Apr 18	Apr 20; fever (38.0°C), chills, headache, vomiting, muscle aches, sore throat, sensitivity to light; nearly 1 wk		May 5; fever (39.8°C), headache, nausea, vomiting, photophobia, poor concentration, blurred vision, tremor; improvement after 8 d	May 8	Pos		Pos	Pos	Neg
2, 59/F	Apr 18	Apr 20; fever (<38.6°C), chills, malaise, loose stools; 5 d		May 3; fever (38.5°C) for 6 d, headache, nausea, confusion, visual disturbances, tremor; marked improvement after 12 d	May 8	Pos		Pos	Pos	Neg
3, 32/M	Apr 17, 18, 20	Apr 20; fever (39.5°C); chills, fatigue, muscle pain; 4 d		Not ill	May 15	Pos		Pos	Pos	Neg
					Jun 6	Pos		Pos	Pos	ND
4, 28/M	Apr 18	Not ill		Not ill	May 15	Neg		Pos	Pos	Neg
					Jun 6	Neg		Pos	Pos	ND

*Except for patient 4, no patients were vaccinated against tick-borne encephalitis. For patients 1 and 2, the incubation period was 2 d; for patient 3, the incubation period was 2–3 d. Patient 1 refused hospitalization and was treated as an outpatient; patient 2 was hospitalized for 8 d; patient 3 did not seek medical care. TBEV, tick-borne encephalitis virus; NT, neutralization test; rRT-PCR, real-time reverse transcription PCR; Pos, positive; Neg, negative; ND, not done.

Patient 3 did not seek medical care. Patients 1 and 2 were examined in the emergency department of the local general hospital on April 20. Laboratory test results were in the reference range, with the exception of mild leukopenia in both patients and mildly elevated liver enzyme levels for Patient 1. TBE was not suspected at that time. All 3 patients recovered in <1 week. Patient 3 remained well, but a second phase of disease developed in Patients 1 and 2 approximately 14 days after the milk was consumed. The second phase was characterized by high fever, headache, nausea (and vomiting in Patient 1), tremor, and mild disturbances of concentration and consciousness. Results of cerebrospinal fluid laboratory tests for Patient 2 revealed abnormalities consistent with aseptic meningitis (reference values are in parentheses): leukocytes 29 × 10^6^/L (<5 × 10^6^/L), neutrophilic granulocytes 9 × 10^6^/L (<5 × 10^6^/L), lymphocytes 20 × 10^6^/L (<5 × 10^6^/L), protein concentration 0.39 g/L (0.15–0.45 g/L), glucose concentration 3.27 mmol/L (2.5–3.9 mmol/L). Patient 1, who refused lumbar puncture diagnostic testing and hospitalization, was treated as an outpatient. The course of disease in Patients 1 and 2 was moderately severe, and the outcome was favorable. A detailed epidemiologic history revealed that none of the 3 patients recalled a recent tick bite and that Patients 2 and 3 consumed raw goat milk rather often, believing it was healthful.

For all 3 patients, TBEV infection was confirmed by 1) ELISA (Enzygnost Anti-TBE/FSME Virus [IgG, IgM]; Siemens, Marburg, Germany) demonstrating specific IgM and IgG against TBEV in serum and by 2) the presence of neutralizing antibodies against TBEV. Real-time reverse transcription PCR of serum samples did not detect TBEV RNA (Table) (*8*).

The fourth person, who remained healthy, was previously vaccinated against TBE. He received his basic vaccination (3 doses) during 1995–1996, the first booster dose in 2000, the second in 2005, and the third in 2010. Serologic test results showed the absence of specific IgM and high levels of specific IgG. An antibody concentration of 912 U/mL in the first serum sample, obtained 27 days after he consumed raw goat milk, and of 672 U/mL in the second serum sample, obtained 3 weeks later, together with a high relative avidity index (85%), suggested a recent booster response.

Patient 3, the owner of a small farm with 9 sheep and 9 goats, including the goat whose milk was consumed, consented to a virologic investigation of serum, blood, and milk samples from his farm animals. By using an indirect immunofluorescent assay, we detected TBEV-specific antibodies in 5 of 9 goat serum samples (titer range 20–1,280) and in 1 of 4 goat milk samples. All samples from sheep were seronegative for TBEV. Quantitative real-time reverse transcription PCR for TBEV was performed on all serum and blood samples and on 4 goat milk samples (*9*). TBEV RNA was detected in serum (1.50 × 10^3^ RNA copies/mL) and milk (1.88 × 10^5^ RNA copies/mL) of the goat whose milk was consumed, confirming the source of infection. TBEV RNA was not detected in samples from the other farm animals.

## Conclusions

Our investigation of illness among 3 of 4 persons who consumed TBEV-infected raw goat milk revealed that all 4 persons were infected with the virus. Febrile illness developed in 3 of the 4 persons 2–3 days after the milk was consumed; the fourth person, who had been vaccinated against TBE, remained healthy. The course of the illness was biphasic in 2 of the 3 symptomatic persons: leukopenia (a characteristic finding for the initial phase of TBE) was present during the initial phase, and the second phase was clinically indicative of meningoencephalitis. Even though 1 of these 2 patients received immunosuppressive therapy because of renal transplantation, the course of the disease was only moderately severe, and the outcome was favorable. A prospective clinical study of patients in the initial phase of TBE who were monitored for the appearance of the second, encephalitic phase of the disease, showed that an abortive form of TBE (i.e., an isolated initial phase not followed by the meningoencephalitic phase), as seen in the third patient in our study, is a rare event (*10*).

After a tick bite, the incubation period for TBE is a median of 8 days (range 4–28 days) (*2*). The incubation period can be shorter for exposure by the alimentary route (*5*) and was found to be only 2 days in the patients in our study. These findings might suggest that drinking TBEV-infected raw milk (colostrum) may result in TBE within a shorter incubation period than when TBE is associated with consumption of infected milk products (e.g., cheese). Therefore, short incubation should not be an exclusion criterion for the diagnosis of TBE, but in proven cases of TBEV infection, a short incubation period is likely a clue for alimentary transmission of TBEV.

Previously reported TBE outbreaks caused by alimentary transmission of TBEV lack definitive evidence of the virus having been present in milk or dairy products. However, in our study of 4 TBEV-infected persons, the source of infection was proven by direct demonstration of TBEV RNA and a corresponding virus load (concentration) in serum and milk samples from the goat whose milk was consumed. The outbreak described herein could have been avoided if the milk had been pasteurized or boiled before consumption or if the persons who became ill had been protected by vaccination, as was the fourth person who drank the TBEV-infected raw milk but did not become ill.

The increasingly fashionable natural lifestyle encourages the consumption of raw milk and products made of unpasteurized milk. Even though alimentary transmission of TBEV is rare, the risk of such exposures could be reduced through education campaigns that encourage persons to consume only milk that has been boiled or pasteurized and only dairy products made from pasteurized milk. In addition, TBE vaccination, which effectively protects against tick bite–associated and dairy product–associated TBEV transmission, should be encouraged in areas where TBEV is highly endemic.
